# Left-Sided Destroyed Lung With Severe Pulmonary Arterial Hypertension as a Consequence of Recurrent Pulmonary Tuberculosis

**DOI:** 10.7759/cureus.56870

**Published:** 2024-03-25

**Authors:** Varun Tiwari, Pankaj Wagh

**Affiliations:** 1 Respiratory Medicine, Jawaharlal Nehru Medical College, Wardha, IND

**Keywords:** pneumonectomy, conservative treatment, pulmonary arterial hypertension, detroyed lungs, pulmonary tuberculosis

## Abstract

Pulmonary tuberculosis is an infection caused by *Mycobacterium tuberculosis*, which is an obligate aerobic microbe. Tuberculosis is a multisystemic disease that can attack the respiratory system, genitourinary system, central nervous system, gastrointestinal system, and the skeletal framework of the body. However, the most commonly affected system is the respiratory system (pulmonary tuberculosis). Tuberculosis is an ancient infection that affects millions of people every year, and even after adequate treatment, it is associated with significant morbidity and mortality, which can be attributed to reinfections, complications, extrapulmonary spread, and the long-term effects of tuberculosis on the lungs, leading to various restrictive and obstructive diseases. One of the most hazardous sequelae of pulmonary tuberculosis is the destroyed lung, which is predominately seen in the culminating stage of progressive disease or after reactivation of the disease. Here we present the case of a 46-year-old female patient who presented with complaints of breathlessness, cough with expectoration, and chest pain. With a history of recurrent tuberculosis infections and appropriate antituberculosis treatment for 30 years, the primary infection was recognized at 16 years of age. On examination, the patient was suspected to have developed fibrosis of the left lung, which, on radiological investigation, was confirmed to be a case of a destroyed left lung because of a recurrent tuberculosis infection. The patient was given symptomatic treatment along with broad-spectrum antibiotic therapy.

## Introduction

Tuberculosis is undoubtedly a chronic lethal infection that is mainly caused by the *Mycobacterium tuberculosis* complex, which can impact any body part, with the lungs being the most frequently affected organ. Usually, an open case of pulmonary tuberculosis is the source of infection. The direct inhalation of aerosolized bacilli present in the droplet nuclei of expectorated sputum is the mode of infection. The initial infection occurs in the alveoli and later develops into a Ghon focus, which can spread to regional lymph nodes, then known as the Ghon complex, which can then progress into an open or closed cavity with a fibrous rim around an inner pool of liquefaction and caseous necrosis. This can further progress to lung fibrosis and lung destruction [1]. Pulmonary tuberculosis can be classified as primary tuberculosis, which is the primary infection in a host, or secondary tuberculosis, which can be due to endogenous reactivation (reactivation of latent infection) or exogenous reinfection [2]. It typically presents with an evening rise in fever, weight loss, night sweats, cough with expectoration, and hemoptysis [3]. Pulmonary tuberculosis can lead to a wide range of complications, including hemoptysis, lung cavity, spontaneous pneumothorax, bronchiectasis, bronchogenic carcinoma, empyema, extensive lung destruction, necrosis, and gangrene [4].

Destroyed lung is a term accepted now in the context of a combination of parenchymal and pleural lung destruction with single or multiple cavitations along with decreased lung volume, bronchiectasis, and mediastinal herniation to the ailing side [5]. This condition can arise due to various pulmonary diseases, particularly infectious diseases of the respiratory system, but is mostly associated with one of the rare long-term effects of tuberculosis. Destroyed lung post-tuberculosis can present a syndrome comprising pulmonary cavitations, pleural and parenchymal fibrosis, loss of lung volume, cystic bronchiectasis, contralateral lung emphysema, unilateral extensive lung parenchymal abnormalities, contralateral tracheal, and mediastinal shift to the diseased side, which can be identified radiologically as mediastinal herniation [6].

## Case presentation

A 46-year-old female patient presented to the outpatient department of respiratory medicine with complaints of cough with expectoration, mucoid consistency, and chest pain for three days. She also complained of increased breathlessness over the past three days, which, as per the Modified Medical Research Council grading, had increased from grade II to grade IV. The patient had a history of multiple hospital admissions due to similar complaints since 2019. Upon further inquiry, the patient revealed that she had contracted pulmonary tuberculosis for the first time at the age of 16 years, for which she underwent antitubercular therapy for six months. Subsequently, at the ages of 22 and 30, she underwent antitubercular therapy for three and two months, respectively. However, during both of these episodes, she failed to follow up at the hospital or adhere to the antitubercular regimen, resulting in an incomplete recovery and recurrent infections. The most recent episode occurred at the age of 45, when she underwent antitubercular therapy for 10 months, by which time fibrosis had developed in her left lung. Notably, there was no history of drug-resistant tuberculosis. In addition to her history of tuberculosis, the patient had been diagnosed with hyperthyroidism for three years and was taking Neo-Mercazole 5 mg once a day. She also had hypertension for one year and was prescribed telmisartan 40 mg once a day. Notably, there was no history of influenza or pneumococcal vaccinations, nor was there any familial or contact history of tuberculosis.

The patient underwent examination under appropriate conditions to check for positive clinical findings. She was afebrile to touch, with a pulse rate of 90 beats per minute, a blood pressure reading of 130/90 mmHg, and an oxygen saturation of 95% on room air, which decreased to 91% on walking. Bilateral lower limb pitting edema was noted during the general examination. No signs of pallor, icterus, cyanosis, clubbing, or lymphadenopathy were observed. Upon inspection, reduced chest wall movements were noted on the left side. Palpation revealed a shift of the trachea toward the left side and decreased tactile vocal fremitus on the left anterior, lateral, and posterior chest walls. Percussion revealed an impaired note on the left chest wall. Auscultation revealed tubular breath sounds over the left chest wall along with bilateral crepitations, predominantly on the left side.

**Figure 1:**
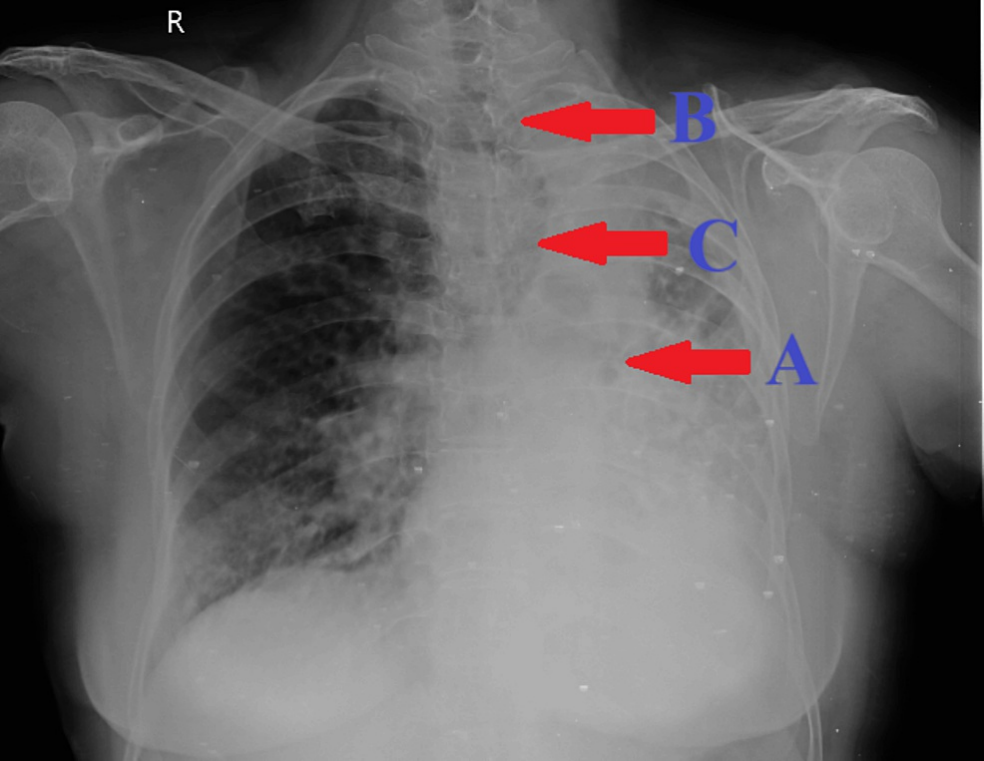
A chest X-ray in PA view displaying (A) extensive destruction of the parenchyma of the left lung; (B) tracheal and mediastinal shift toward the diseased side; and (C) herniation of a portion of the right lung into the left hemithorax PA, posteroanterior

Further assessment included a chest X-ray in posteroanterior (PA) view, which revealed extensive destruction of the parenchyma of the left lung with an ipsilateral tracheal and mediastinal shift toward the diseased side. Additionally, herniation of a portion of the right lung into the left hemithorax was observed (Figure 1). A two-dimensional echocardiography report indicated severe pulmonary arterial hypertension. Routine blood investigations showed no significant abnormalities (Table 1). Sputum samples sent for acid-fast bacilli staining were negative.

**Table 1:** Routine blood investigations

Investigations	Patient values	Reference values
Hemoglobin	11.9 grams/deciliter	11.5–16.5 grams/deciliter
Total erythrocyte count	4.52 million per cubic millimeter	3.8–5.8 million per cubic millimeter
Total leucocyte count	10,900/microliter	4,000–11,000/microliter
Total platelet count	264,000/microliter	150,000–400,000/microliter
Serum urea	42 milligrams/deciliter	20–40 milligrams/deciliter
Serum creatinine	1.5 milligrams/deciliter	0.6–1.2 milligrams/deciliter
Serum bilirubin	0.6 milligrams/deciliter	0.3–1.3 milligrams/deciliter
Serum albumin	4.0 grams/deciliter	3.5–5.5 grams/deciliter
Serum glutamic oxaloacetic transaminase	21 units/liter	12–38 units/liter
Serum glutamate pyruvate transaminase	15 units/liter	7–41 units/liter
Thyroid-stimulating hormone	0.508 microunits/milliliter	0.4–5 microunits/milliliter
Random blood sugar	149 milligrams/deciliter	<140 milligrams/deciliter

The patient was prescribed broad-spectrum antibiotics along with glucocorticoids, diuretics, and phosphodiesterase 5 inhibitors. Additionally, she underwent nebulization with budesonide, ipratropium, and levosalbutamol. The patient was advised to continue her medication regimen for hyperthyroidism and hypertension as previously prescribed. Furthermore, she was recommended to receive influenza and pneumococcal vaccinations. Following symptomatic relief, the patient was discharged from the hospital.

## Discussion

Tuberculosis is a fatal chronic infection worldwide that causes enormous morbidity and mortality every year, and it becomes even more important in the context of the Indian population. Early diagnosis and prompt treatment do not suffice to provide complete relief for every patient. The National Tuberculosis Elimination Programme (NTEP) of India employs well-defined standards to treat the condition [7]. However, even after completing the antituberculosis treatment regime, there are chances that tuberculosis might continue to impact patients with its short-term or long-term effects or in the form of reinfections, as seen in this case. The prolonged effects or complications can be attributed to irrevocable lung damage because of a chronic infection. Among those complications, a common but extremely fatal complication is the destroyed lung syndrome, which can be defined as the extensive deterioration of lung parenchyma with reduced lung function [8]. Various conditions can lead to the destruction of the lungs, like bronchial stenosis, bronchovascular fistula, bronchiectasis, invasive opportunistic infections, aspergillosis, fibrosing mediastinitis, and emphysema [6,9].

A destroyed lung is found to be extremely detrimental as it can lead to complications that are often fatal, such as septicemia, massive hemoptysis, empyema, respiratory insufficiency, secondary fungal infections, and a left-right shunt. The left-right shunt can lead to increased pulmonary pressure and pulmonary arterial hypertension, which could present as dyspnea, cough, chest pain, palpitations, edema, and cyanosis [10]. The diagnosis of a destroyed lung can be established by using various radiological modalities, such as chest radiography, computed tomography of the chest, and bronchography. On chest X-ray PA view, the presence of lung parenchymal destruction along with ipsilateral mediastinal and tracheal shift and contralateral lung herniation is confirmatory of a destroyed lung [11]. There is no definite treatment protocol or guideline for the management of destroyed lungs. However, conservative treatment meant to provide symptomatic relief to the patient can be put to use. This includes the use of long-acting beta-2 agonists or long-acting muscarinic antagonists, along with corticosteroids, oral mucolytics, and broad-acting antibiotics. Surgical management can be used as a definitive treatment that involves a procedure with high risk called pneumonectomy that is used to resolve or avert the complications related to destroyed lungs [12]. Pulmonary arterial hypertension associated with destroyed lungs can be managed with phosphodiesterase 5 inhibitors, endothelin receptor antagonists, calcium channel blockers, prostacyclin agonists, and soluble guanylate cyclase stimulators. The treatment for pulmonary arterial hypertension largely remains palliative in nature [13].

## Conclusions

Pulmonary tuberculosis can be managed effectively with early diagnosis and appropriate treatment by antitubercular drugs given under the directly observed therapy short course. However, lung function deterioration is known to happen following pulmonary tuberculosis due to certain irreversible changes in the lung that can lead to complications such as a destroyed lung, which can result from reinfections, as in this case. And so, under the NTEP, there needs to be greater awareness of the symptoms, early diagnosis, advantages of suggested treatment, cost-effectiveness of treatment, free and widely accessible antituberculosis therapy, and the positive roles played by all of these in preventing destroyed lungs and other complications that have a detrimental effect on the overall outcome.

## References

[1] Kasper D, Fauci A, Hauser S, Longo D, Jameson J, Loscalzo J (2015). Harrison's Principles of Internal Medicine. https://cir.nii.ac.jp/crid/1130573781693502243.

[2] Cardona PJ (2016). Reactivation or reinfection in adult tuberculosis: is that the question?. Int J Mycobacteriol.

[3] Alvarez S, Shell C, Berk SL (1987). Pulmonary tuberculosis in elderly men. Am J Med.

[4] Allwood BW, Byrne A, Meghji J, Rachow A, van der Zalm MM, Schoch OD (2021). Post-tuberculosis lung disease: clinical review of an under-recognised global challenge. Respiration.

[5] Patil S, Narkar S, Raka V, Dahiphale J, Choudhari S, Gondhali G (2023). 'Destroyed lung’ as post tuberculosis sequel: a preventable stigma of ‘disease of concern’ of millennium!. Saudi J Med.

[6] Yadav S (2023). Destroyed lung syndrome in a young Indian male: a case report. Cureus.

[7] Khatri GR, Frieden TR (2002). Controlling tuberculosis in India. N Engl J Med.

[8] Osarenkhoe J, Aiwuyo H, Aisosa O, Ejiroghene U (2022). Destroyed lung syndrome: a review of 31 published cases. Open J Respir Dis.

[9] Owen RM, Force SD, Pickens A, Mansour KA, Miller DL, Fernandez FG (2013). Pneumonectomy for benign disease: analysis of the early and late outcomes. Eur J Cardiothorac Surg.

[10] Han D, Lee HY, Kim K, Kim T, Oh YM, Rhee CK (2019). Burden and clinical characteristics of high grade tuberculosis destroyed lung: a nationwide study. J Thorac Dis.

[11] Palmer PE (1979). Pulmonary tuberculosis — usual and unusual radiographic presentations. Semin Roentgenol.

[12] Ruan H, Liu F, Li Y (2022). Long-term follow-up of tuberculosis-destroyed lung patients after surgical treatment. BMC Pulm Med.

[13] Mayeux JD, Pan IZ, Dechand J (2021). Management of pulmonary arterial hypertension. Curr Cardiovasc Risk Rep.

